# Implementation of a Quality Improvement Role for Unlicensed Assistive Personnel and Effects on Infection Prevention

**DOI:** 10.1017/ash.2021.144

**Published:** 2021-07-29

**Authors:** Natalie Schnell, Lauren DiBiase, Amy Selimos, Lisa Stancill, Shelley Summerlin-Long, Emily Sickbert-Bennett

## Abstract

**Background:** Care bundles comprise evidence-based practices and interventions that are easily and consistently implemented while improving patient outcomes. As patient acuity and task overload continue to increase, infection prevention bundle and process measure compliance and data collection may become a lower priority for registered nurses (RNs). In early 2019, a certified nursing assistant (CNA) began full-time quality liaison work on a 53-bed inpatient adult oncology unit at UNC Medical Center to provide targeted compliance data collection and to correct deficits in real time when possible and within the appropriate scope of practice. **Methods:** The quality liaison CNA is highly motivated, with a relevant clinical background and effective communication skills. After conducting a gap analysis, the unit developed specific responsibilities for several areas of quality improvement, including infection prevention. In addition to rounding on all patients daily, the quality liaison (1) performs direct patient care tasks like Foley catheter care, (2) conducts patient education on topics such as chlorhexidine gluconate treatments, (3) performs all relevant process measure audits, and (4) easily relays missed or needed care to RNs with a door sign created as part of this initiative. High-risk findings, such as a loose central-line dressing, prompt immediate communication to the RN, with follow-up and escalation when necessary. **Results:** Patients and staff received the quality liaison well, and the increased attention to care bundle components and auditing ensured consistent, evidence-based care along with accurate and reliable data collection. Compared to the previous calendar year, the number of central-line audits on the unit increased by >1,400 by the end of 2019. Patient outcomes improved, and during 1 fiscal year, the unit achieved rate reductions between 40% and 55% for central-line–associated bloodstream infections, catheter-associated urinary tract infections, and healthcare-associated *C. difficile* infections. Staffing and logistical challenges imposed by the COVID-19 global pandemic have hampered this work because the quality liaison was redeployed to direct patient care intermittently. Correspondingly, from July to October 2020, the same infection rates increased between 30% and 353%. **Conclusions:** Having a designated quality liaison is an effective means to achieving quality improvements while remaining an integral member of the patient care team. As staffing has improved on this unit, the quality liaison has refocused efforts, and infection rates are beginning to improve. Given the success of the quality liaison role in improving quality outcomes on this unit, the hospital is exploring expansion of this model to additional units.

**Funding:** No

**Disclosures:** None

